# Aloe-Emodin Overcomes Anti-Cancer Drug Resistance to Temozolomide and Prevents Colony Formation and Migration in Primary Human Glioblastoma Cell Lines NULU and ZAR

**DOI:** 10.3390/molecules28166024

**Published:** 2023-08-11

**Authors:** Sabrina Staffieri, Veronica Russo, Maria Antonietta Oliva, Marika Alborghetti, Miriam Russo, Antonietta Arcella

**Affiliations:** 1 IRCCS Istituto Neurologico Mediterraneo NEUROMED, Via Atinense 18, 86077 Pozzilli, Italy; sabrina.staffieri@neuromed.it (S.S.); veronoica.russo1@unicampania.it (V.R.); mariaantonietta.oliva@neuromed.it (M.A.O.); 2 Department of Neurosciences, Mental Health and Sensory Organs (NESMOS), Sapienza University of Rome, 00185 Rome, Italy; marika.alborghetti@uniroma1.it; 3 Dipartimento di Bioscienze e Territorio, Università Degli Studi del Molise, Contrada Fonte Lappone, 86090 Pesche, Italy; m.russo15@studenti.unimol.it

**Keywords:** glioblastoma (GBM), aloe-emodin (AE), temozolomide (TMZ), autophagy, apoptosis, *MGMT*

## Abstract

Glioblastoma, the most dangerous and aggressive type of CNS tumor, appears resistant to many chemotherapy drugs. In the patient-derived glioma cell lines NULU and ZAR, which exhibit drug-resistant phenotypes, we investigated the effect of combined AE (Aloe-emodin) and TMZ (temozolomide) and found a significant additive inhibitory effect on cell growth and a promising cytotoxic effect on both cell lines compared to treatment with single agents. We also examined the effect of combined AE and TMZ treatment on the drug-resistance protein MGMT. The results suggest that using AE combined with traditional drugs restores drug resistance in both primary resistant cell lines (NULU and ZAR). Furthermore, migration assays and scratch tests showed that the combined use of AE and TMZ can slow down the colony formation and migration of glioblastoma cells. These convincing results suggest that AE could be a natural adjuvant agent to potentiate the effects of traditional drugs (TMZ) and overcome drug resistance in glioblastoma cells.

## 1. Introduction

Glioblastoma multiforme (GBM), which most commonly affects adults, is the most harmful and common brain tumor. It is a malignant tumor that develops rapidly, broadly, and infiltratively. It is believed to arise from neural stem cells (NSCs) in the subventricular zone (SVZ) of the adult human brain. Factors such as excessive angiogenesis, cellular heterogeneity, and the presence of tumor stem cells, which can proliferate and generate glial neoplastic cells, contribute to a poor prognosis [1,2]. The median survival time for patients with this type of tumour after surgery and successful radiotherapy or chemotherapy is 14 months [3], with a 2-year survival rate of 2% [4]. The standard protocol calls for intensive radio-chemotherapy in the first month following surgery, followed by an additional TMZ cycle [5]. The tumor frequently relapses over a period that varies from patient to patient because treatment frequently cannot completely control it. Although there has been significant progress in our comprehension of the molecular mechanisms underlying the growth and development of GBM, more investigation into the genetic mechanisms underlying tumor progression is still needed [6]. In order to achieve this goal and discover a novel, promising in vitro therapy that will successfully translate in vivo, patient-derived glioma cell lines were established in our laboratory. These cell lines faithfully maintain the phenotypic and molecular characteristics of the tumor from which they originate. Additionally, these in vitro cultures are used for research on drug transport, the cytotoxicity of natural chemicals, the validation of novel experimental compounds, and the development of target therapeutics [7]. Several molecular markers are used to gauge the prognosis of GBM patients. The methylation status of one of them, O6-methylguanine–DNA methyltransferase (MGMT), is regarded as a marker for a good prognosis [8,9]. The MGMT gene, located at the 10q26 locus, encodes the MGMT enzyme. The protein MGMT repairs DNA damage from alkylating chemicals [9,10]. Methylation of the *MGMT* DNA repair gene promoter inhibits MGMT DNA repair protein expression, resulting in a chemosensitive phenotype and longer survival in GBM patients treated with alkylating agents [10,11]. Studies have shown that adjuvant radiation and chemotherapy with TMZ double survival in GBM cases with MGMT gene promoter methylation compared to radiotherapy alone [12]. The eradication of GBM is difficult, if not impossible, due to the intrinsic molecular variability of GBM and the variability of the tumor’s response to the alkylating drug TMZ. Therefore, funding for new research is required to identify adjuvant substances that can kill GBM cells in various ways, and it would be intriguing to discover substances that can control drug resistance. It has become evident over the past 10 years that bioactive phytochemicals have minimal to no toxicity and can effectively treat a variety of cancers, including brain tumors [13]. Our research focuses on the natural bioactive phytochemical AE and its effect on GBM cell drug resistance and migration. Fresh AE-containing leaves have various groups of chemical compounds, such as glycoproteins, polysaccharides, anthraquinone derivatives, vitamins, minerals, amino acids, and many more, which show multidirectional therapeutic action [14].

Herbs containing AE have been widely used as traditional medicines in many countries, especially in East Asia. Over the past 3 years, there have been many reports of the anti-cancer and anti-inflammatory effects of AE. AE (1,8-dihydroxy-3-hydroxymethyl-anthraquinone) is a naturally occurring anthraquinone derivative that is present in several popular Chinese medicinal herbs, including *Polygonum multiflorum Thunb*, *Cassia occidentalis*, *Rheum palmatum* L., *Aloe vera*, and *Rheum palmatum* [15]. AE might be a good therapeutic option for the prevention and treatment of many diseases, including osteoarthritis, pancreatic cancer, breast cancer, hepatocellular carcinoma, and Alzheimer’s disease [16,17]. In a previous study, we used AE (20 μM and 40 μM) to demonstrate the effects on U87MG human GBM cells, both in vitro and in vivo. The results showed that AE significantly reduces U87MG cell proliferation [18]. 

This study investigates how AE, a substance derived from natural aloe leaves, can regulate and overcome drug resistance in primary GBM cell lines with a drug-resistance phenotype (unmethylated MGMT: NULU and ZAR). AE was combined with TMZ and used to treat NULU and ZAR cells to monitor the levels of the MGMT protein, one of the key proteins contributing to drug resistance in GBM. The effect of AE on the proliferation of primary glioblastoma cell lines NULU and ZAR was also evaluated, in addition to the ability of AE to prevent colony growth and delay cell migration, which are the primary causes of GBM recurrence.

## 2. Results

### 2.1. Impact of AE on Primary GBM Cell Lines NULU and ZAR, Both as a Single Agent and in Combination with TMZ

**Figure 1:**
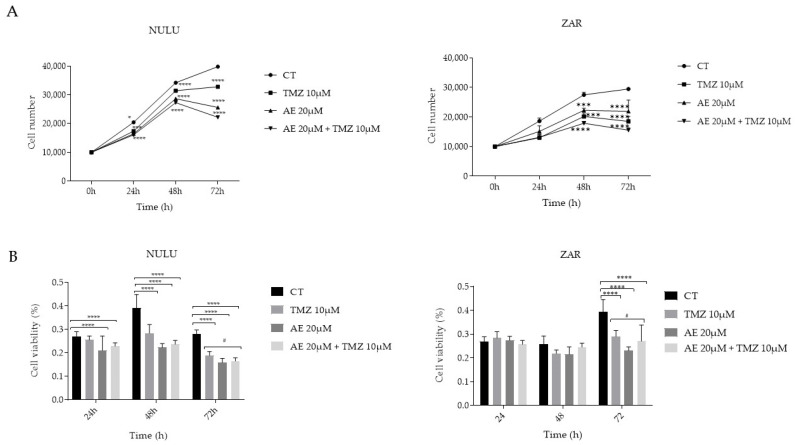
Effect of 20 μM AE alone and in combination with 10 μM TMZ on cell growth and viability in primary GBM lines, NULU and ZAR. (**A**) Effect of AE, as a single agent and in combination with TMZ, on the number of NULU and ZAR primary GBM cells exposed daily to TMZ 10 μM, AE 20 μM, and AE 20 μM and TMZ 10 μM for 24, 48, and 72 h; (**B**) cytotoxic effects of daily exposure to TMZ 10 μM, AE 20 μM, and AE 20 μM and TMZ 10 μM for 24, 48 and 72 h on the cellular metabolism of NULU and ZAR GBM cells. For all experiments, the reported values are the mean ± SEM of 3 independent determinations: one-way ANOVA test, Dunnett’s multiple comparison test, *p*-value < 0.05 is significant. According to the GraphPad Prism 7 software, * *p*-values between 0.01 and 0.05; *** *p*-values between 0.0001 and 0.001 (compared to the control); a **** *p*-value < 0.0001 (compared to the control); a # *p*-value between 0.01 and 0.05 (compared to TMZ).

The primary human glioblastoma lines NULU and ZAR, selected based on the methylation profile (unmethylated MGMT) and therefore resistant to treatment with alkylating drugs, were exposed to a combination of AE 20 μM and TMZ 10 μM for 24, 48, and 72 h. The AE concentration was evaluated by IC50 (IC50 = 19.74 ± 0.14, as shown in Figure S1). The potential adjuvant effect of AE with standard TMZ therapy was then compared with the two single treatments and a control treated with DMSO alone as a control vehicle. The effects of the combination therapy were evaluated on cell growth and viability. As shown in Figure 1A, AE showed a significant inhibitory effect on the growth of GBM cell lines NULU and ZAR starting after 24 h of exposure, confirming the inhibitory growth effect of AE on the primary lines of GBM. Although the low efficacy of 10 μM TMZ in controlling the growth of NULU and ZAR lines is perfectly related to the methylation of the *MGMT* promoter, concomitant treatment with the two molecules significantly increased the effect of TMZ alone, inhibiting the replicative potential of NULU cells and ZAR as early as 24 h after treatment. At 72 h of treatment, the combined effect of AE 20 μM and TMZ 10 μM was particularly significant, showing a reduction of 65% against the treatment with TMZ alone and 75% against the control in the ZAR cell line. Similarly, in the NULU cell line, the combined treatment of AE 20 μM and TMZ 10 μM at 72 h determined a reduction in cell growth of 48% compared to the treatment with only TMZ 10 μM and 58% compared to the control. From the metabolic point of view in the MTT assay (Figure 1B), the effect of AE is more marked on the NULU cell line, which, starting from 24 h of treatment, shows a reduction in the mitochondrial dehydrogenase activities of 23%, up to 43% after 72 h of exposure to AE 20 μM. Similarly, to what was previously observed on the U87MG commercial GBM line, the effect of AE 20 μM is particularly evident after 72 h of treatment, as also shown by the results obtained on the primary ZAR line. Of particular relevance is the effect exerted by AE 20 μM on improving the cytotoxicity induced by TMZ 10 μM at 72 h of exposure in both cell lines. These data suggest a possible involvement of AE in the mechanisms of drug resistance that overcome the underlying fatal response of GBMs expressing the MGMT enzyme. In fact, the inhibitory effect of combined AE and TMZ on cell growth is significant compared to the effect of TMZ alone (*p*-value between 0.01 and 0.05). However, the interpretation of the MTT experiment is different; the effect of AE in combination with TMZ is not additive in toxicity, showing and substantiating that AE is nontoxic (Figure 1B). AE could inhibit the growth of glioblastoma cell lines via other interesting mechanisms.

### 2.2. Analysis of the Expression of the MGMT Protein in the NULU and ZAR Primary GBM Lines Treated with TMZ and AE Alone/in Combination

**Figure 2:**
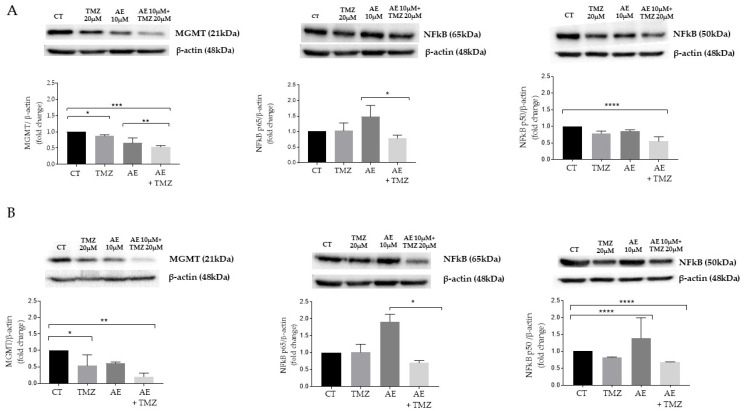
Examination of the expression of the MGMT protein and the NF-κB transcription factor in the primary GBM lines NULU and ZAR after exposure to AE and TMZ alone or in combination. Western blot analysis of MGMT protein and transcription factor NF-κB p65 and p50 subunits of NULU (**A**) and ZAR (**B**) cells were treated long-term (72 h) with 20 μM AE and 10 μM TMZ, singly and in combination. Controls were treated with 0.3% DMSO. Normalization was performed with the β-actin. The densitometric analysis of protein levels represents the mean ± SEM of three individual determinations: one-way ANOVA test, Dunnett’s multiple comparison, test *p*-value < 0.05 is significant. According to GraphPad Prism 7 software, * *p*-values between 0.01 and 0.05; ** *p*-values between 0.001 and 0.01 (with respect to the control); *** *p*-values between 0.0001 and 0.001 (compared to the control); **** *p*-values < 0.0001 (compared to the control).

GBM primary cell lines, derived from NULU and ZAR patients, exhibit an unmethylated profile of the *MGMT* gene promoter and, consequently, a drug-resistance phenotype to conventional TMZ chemotherapy. Therefore, evaluating possible future clinical applications, we studied the expression of MGMT protein in NULU and ZAR primary GBM lines exposed, alone and in combination with 10 μM TMZ, to 20 μM AE for 72 h. As can be seen in Figure 2A,B, the Western blot analysis of MGMT protein expression, normalized on β-actin protein level expression, revealed the ability of AE 20 μM to reduce the expression of this protein in both primary GBM lines. The even more significant *datum* derives from the combination AE 20 μM and TMZ 10 μM, which determines a significant reduction (*p* < 0.05) of the expression of the MGMT protein compared to the control (treated with DMSO) and compared to the single treatments with TMZ 10 μM and AE 20 μM. The reduction in MGMT protein expression could be the key mechanism underlying the overcoming of drug resistance induced by AE.

### 2.3. Analysis of the Expression of the Transcription Factor NF-κB in Primary GBM Lines Exposed to Treatment with AE, TMZ, and in Combination

Among the several transcription factors known to regulate MGMT protein expression, the activity of the factor NF-κB is well known [19,20]. In order to determine a potential correlation between the reduction in MGMT protein expression and the transcription factor NF-κB, we assessed the Western blot of protein lysates from the NULU and ZAR lines treated with TMZ and AE. Figure 2A,B show the expression of the two subunits that compose the transcription factor NF-κB, p50, and p65. In NULU and ZAR lines, which express MGMT, the combined treatment with AE 20 μM and TMZ 10 μM significantly reduced the expression of the p65 and p50 subunits compared to the untreated control and samples subjected to single treatment (AE alone or TMZ alone). These results suggested that the downregulation of MGMT may be directly related to reduced NF-κB transcriptional activity. This downregulation could be functionally translated with a higher TMZ sensitivity.

### 2.4. NULU mRNA Levels of BRCA1, ATM, and DNA-PK after AE and TMZ Treatment

**Figure 3:**
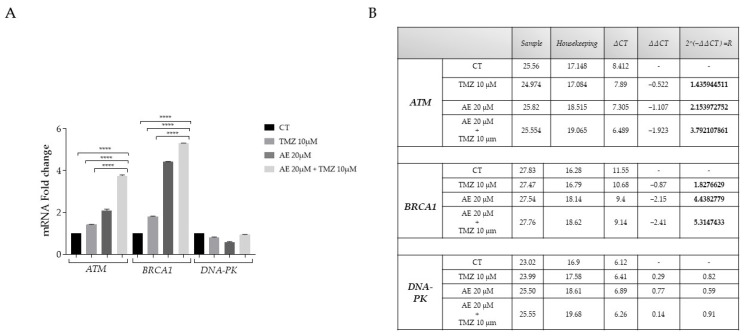
Real-time PCR of *ATM*, *BRCA1*, and *DNA-PK*. *ATM*, *BRCA1*, and *DNA-PK* mRNA after treatment with AE 20 μM alone and combined with TMZ 10 μM DNA damage repair genes expression in NULU GBM primary cells were determined by real-time PCR. The total RNA was extracted from the NULU cells after treatment with 20 μΜ AE for 72 h, and RNA samples were reverse transcribed for cDNA and then for real-time PCR, as described in Materials and Methods. The ratios of *ATM*, *BRCA1*, and *DNA-PK* mRNA compared to *GAPDH* (**A**) are presented; **** *p*-values < 0.0001 (compared to the control). Data represent the mean ± SEM (**B**).

Temozolomide is an oral alkylating drug that has been given FDA approval for first-line treatment of GBM. TMZ functions by methylating DNA, and damaging DNA inhibits cellular and DNA replication. The cellular response to damage includes more steps than just the DNA repair process by alkylating agents alone, in which MGMT plays a direct role. The DNA repair machinery is actually a complex “network” of functions involving multiple double-stranded repair proteins [21], which in turn activate multiple cell cycle checkpoint proteins. BRCA1, ATM, and DNA-PK are the three most important. ATM is responsible for starting the complex sequence of processes that make up the cellular response to injury, including activating the BRCA1 protein and cell cycle checkpoints; BRCA1 and DNA-PK control the functional complex and related activities to protect genomic integrity [22,23]. Following treatments for 24 h, 48 h, and 72 h with AE, TMZ, and the two substances combined, we measured the mRNA levels of the corresponding genes via real-time PCR. Contrary to our expectations, AE and AE plus TMZ treatment increased the transcription of the examined genes. This observation could be interpreted in terms of a compensatory intrinsic mechanism present in GBM cells (Figure 3). We suggested that the increased transcription of *BRCA1*, *ATM*, and *DNA-PK* might counteract the *MGMT* depletion induced by AE and TMZ treatment of glioblastoma cells. We hypothesized that AE might interfere with the regulation of the complex DNA damage mechanism composed of BRCA1, ATM, and DNA-PK through BAAT1-mediated phosphorylation of BRCA1 and DNA-PK [24].

### 2.5. The Effects of TMZ and AE Adjuvant Therapy on the Clonogenic Capacity of the Primary GBM Lines NULU and ZAR

**Figure 4:**
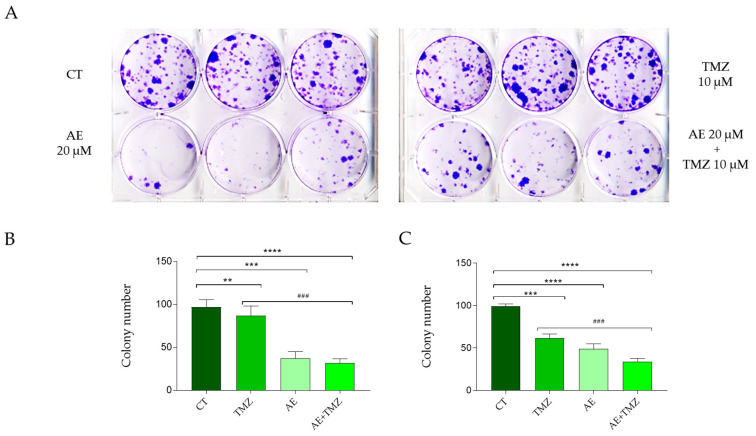
Effect of AE and TMZ on the NULU and ZAR clonogenic potential. (**A**) Representative image of NULU line clonogenic potential assay; (**B**,**C**) primary glioblastoma NULU and ZAR line clonogenic potential and the impact of AE 20 μM alone and in combination with TMZ 10 μM on the capacity to form colonies. For all experiments, the reported values are the mean ± SEM of three independent determinations. One-way ANOVA; *p*-value < 0.05 is significant. According to GraphPad Prism 7 software), ** *p*-values between 0.001 and 0.01 (with respect to the control); *** *p*-values between 0.0001 and 0.001 (compared to the control); **** *p*-values < 0.0001 (compared to the control). ### *p* value (compared to TMZ).

GBMs exhibiting a molecular profile with unmutated *IDH1* and an unmethylated *MGMT* gene promoter tend to recur in most cases, contributing to the poor prognosis of this brain tumor. The most popular in vitro method for determining whether a single cell can self-renew in a colony of 50 or more cells, the colony test, offers an estimation of the possibility that a single cancer cell will be able to cause a tumor recurrence [25]. In this regard, the ability of the cells of the NULU and ZAR primary lines to form colonies was evaluated. As can be seen in Figure 4, both cell lines are capable of forming colonies, and treatment with AE 20 μM significantly reduces the number of colonies compared to controls treated with DMSO. The ability of TMZ to only marginally control clone formation further underlines how this cellular model can be representative of in vitro pathology. The adjuvant effect of AE 20 μM on TMZ 10 μM becomes particularly relevant, the combined effect of which surprisingly reduces the number of colonies compared to the controls and enhances the effect of TMZ. Therefore, the ability of a natural adjuvant substance (AE) to control the clonogenic potential of GBM cells, delaying or inhibiting their recurrence, is a particularly interesting aspect to be proposed as an adjuvant therapy combined with TMZ in treating GBM.

### 2.6. Scratch Test to Assess the Effectiveness of the Combination of AE and TMZ in Preventing the Migration of the Primary GBM Lines NULU and ZAR

**Figure 5:**
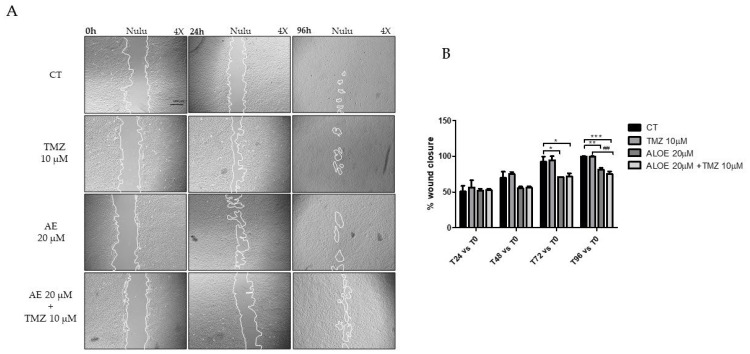
(**A**) Migration of the primary line NULU glioblastoma cells in response to AE 20 μM alone and in combination with TMZ 10 μM. Representative images of the scratch test in the NULU cell line, exposed to treatment with AE 20 μM, single and in combination with TMZ 10 μM at 0, 24, and 96 h, under the Evos FL microscope (4× magnification). Controls were treated with 0.3% DMSO. (**B**) The graph shows the quantification of the scratch test as a percentage of closure of the selected area. For all experiments, the reported values are the mean ± SEM of 3 independent determinations. One-way ANOVA, Bonferroni’s multiple comparison test, and *p*-value < 0.05 are significant. According to GraphPad Prism 7 software, * *p*-values between 0.01 and 0.05; ** *p*-value between 0.001 and 0.01 (with respect to the control); *** *p*-values between 0.0001 and 0.001 (compared to the control), ## *p*-value (compared to TMZ).

Glioblastomas almost inevitably recur in a more resistant-to-therapy state, sometimes at sites distant from the site of origin; therefore, the ability of AE to control the migration of NULU and ZAR GBM cells was evaluated by a “scratch” test, alone and in combination with TMZ. Compared to the controls, the AE 20 μM decrease corresponds to the times of closure of the “scratch” in both cell lines. The percentage of scratch closure, and therefore the ability of NULU and ZAR cells treated with 20 μM AE alone, to migrate and close the selected area, is significantly lower than the treatment with 10 μM TMZ alone and toward the control, starting at 48 h and in particular at 72 h and 96 h post-scratch. In detail, the percentage of scratch closure in the AE-treated NULU cells compared to the control was approximately 70% vs. 92% at 72 h and 81% vs. 100% at 96 h. In the NULU cell line, the effect of the combined treatment, AE 20 μM and TMZ 10 μM, was comparable to the treatment with AE 20 μM alone at 72 h of exposure but more efficient in controlling the closure of the scratch at 96 h of treatment (Figure 5).

Additionally, in the ZAR cell line compared to the control, the result was approximately the same for NULU cells (data are shown in Supplementary Data S1 and S2).

In fact, for both cell lines treated with only AE 20 μM or with AE 20 μM and TMZ 10 μM, the scratch was still open after 96 h of exposure. The inability of TMZ 10 μM to control NULU and ZAR cell migration underscores how adjuvant treatment with AE could reduce the ability of cells to migrate and dare to recur at sites distant from the site of GBM origin.

## 3. Discussion

Despite mainstream therapies, recurrence and drug resistance remain the primary reasons for treatment failure in GBM [19]. The intricacy of this malignant disease and the multiple pathways of treatment resistance contribute to the difficulty of treating it [26]. Drug efflux, hypoxic tumor cell regions, cancer stem cells, DNA damage repair, and miRNAs are some of the factors that contribute to treatment resistance in GBM. New pharmaceutical formulations, such as nanoparticles and viral vectors, as well as new strategies involving the use of monoclonal antibodies, vaccines, and immunotherapy drugs, such as checkpoint inhibitors, will be taken into consideration to help combat drug resistance. Numerous potential treatments aim to address these mechanisms [20]. It is important to pay particular attention and continue studying the newly available evidence on the potential of plant-derived therapies. Over the past decade, bioactive phytochemicals from aloe arborescens have been recognized as having little or no toxicity at all in the treatment of many forms of cancer, including brain tumors [27,28]. In our previous study [18], using the human glioblastoma cell line U87MG, we examined the anticancer effects of AE, an anthraquinone molecule found in the leaves of AE arborescens, and showed the promising antiproliferative effect of AE alone, in vitro, and in vivo, on a mouse xenograft model. However, common commercial GBM cell lines (e.g., U87MG, U251, T98G, and A172) are genetically distant from primary tumors due to the high number of passages in culture, losing the typical heterogeneity of GBM cells. Additionally, the MGMT methylated gene promoter profile of the commercial GBM line U87MG’s cells prevents us from studying drug-resistance mechanisms in vitro. Instead, the primary cell lines selected for this investigation are NULU and ZAR, which have a nonmethylated profile of the MGMT gene promoter and exhibit a drug-resistance phenotype. This cellular model accurately mimics the patient’s tumor in vitro [7], keeping the normal heterogeneity of GBM and enabling us to research the mechanisms underlying TMZ treatment resistance. In this case, the effects of AE 20μM alone and in combination with TMZ 10 μM treatment on cell growth and viability were assessed at 24, 48, and 72 h after exposure to the primary GBM lines NULU and ZAR. The outcomes of the daily cell count and MTT assay revealed that the primary GBM lines NULU and ZAR were also affected by the inhibitory effects of AE 20 μM on cell growth and viability beginning at 24 h of exposure. Particularly pertinent was the stronger inhibitory impact seen when AE 20 μM and TMZ 10 μM were used in combined treatment, which manifested to potentiate TMZ alone. As mentioned previously, molecular characterization of GBM cell lines derived from NULU and ZAR patients revealed the unmethylated profile of the *MGMT* gene promoter and, consequently, a drug-resistance phenotype to standard TMZ chemotherapy. The expression of MGMT may be regarded as the most significant molecular predictor of TMZ resistance and prognosis in gliomas, provided that the cytotoxic effects of the alkylating agent can be mitigated in cells with a high level of endogenous MGMT activity. Furthermore, mounting data highlight the critical function of the NF-κB signaling system in the treatment resistance of GBM. Cytotoxic medications such as taxanes, vinca alkaloids, and topoisomerase inhibitors cause the activation of NF-κB. Similar to this, activation of the DNA damage pathway by alkylating drugs, such as carmustine and TMZ, can also activate NF-κB [20,21,22].

This led us to monitor, using Western blot, the expression levels of the MGMT protein and NF-κB both in the NULU and the ZAR lines after single (TMZ) and combined treatments with AE. The results of the analysis of the expression of MGMT protein showed the presence of MGMT protein in all samples, but highlighted a strong reduction after AE/TMZ combined treatment. Western blot for the transcription factor NF-κB after additive treatment with AE plus TMZ showed a significant signal reduction as well, suggesting the mediation of a potential downregulation in MGMT NF-κB. In fact, a reduced expression of the p50 and p65 subunits of the factor NF-κB in both cell lines of GBM exposed for 72 h is also evidenced in the specific Western blot. For instance, downregulating the NF-κB p50 and p62 homodimers in AE+TMZ-treated GBM cells could be a mechanism to decrease drug resistance in primary GBM cell lines. It is interesting to note that many activities, including the maintenance of cancer stem-like cells, promotion of cancer cell invasion, support for mesenchymal identity, and radiation resistance, have been linked to the decrease in NF-κB activity following treatments, which may regulate other functions [20]. The complex protein pathways that cells use to react to genotoxic stress are referred to as the DNA damage response (DDR). In addition to DNA repair by alkylating chemicals alone, in which MGMT is directly involved, the intricate “network” of events constituting the cellular response to damage extends considerably [29]. A notable illustration of this is how the body responds to highly cytotoxic lesions, such as double-stranded breaks (DSBs). One distinctive aspect of this response is the activation of cell cycle checkpoints. The abrupt arrest of the cell cycle alters numerous physiological processes. In actuality, DNA damage influences the expression of genes as well as the production, degradation, and localization of proteins [30]. This intricate network of phosphorylation-responsive mechanisms is primarily activated by the central protein kinase ATM, with BRCA1 and DNA-PK playing a significant role. Therefore, after treatment with AE, TMZ, and AE and TMZ in combination, we assessed the levels of ATM, BRCA1, and DNA-PK in NULU and ZAR. Surprising results imply that this gene’s mRNA levels rise during treatment as a result of compensatory mechanisms against *MGMT*’s downregulation.

We thought it would be interesting to look into the capacity of AE alone and in combination with TMZ to control the clonogenic potential and migration of NULU and ZAR GBM cells under the same experimental conditions. NULU and ZAR primary GBM cells produced fewer colonies when exposed to AE or TMZ after 15 days; however, a higher inhibitory effect on colony formation was observed when AE and TMZ were combined, evidencing a significant and additive effect when compared to control and single treatments. In addition, the scratch test results also show that synergistic treatment with concomitant TMZ and AE strongly inhibits cell migration. The inability of TMZ alone to regulate clone formation and cell migration highlights the fact that this cellular model is representative of in vitro pathology and shows the adjuvant effect of AE’s potential to delay or inhibit the possibility of tumor recurrence due to the presence of clones in sites distant from the site of origin, two crucial components for effective treatment against GBM.

## 4. Methods

### 4.1. Cell Culture

Primary human GBM cells (NULU and ZAR) were cultured in vitro from brain biopsies of Neuromed neurosurgery patients who gave informed consent to participate in this study. The use of primary cell lines as a model for GBM heterogeneity was approved by the ethics committee on 27 February 2020, and registered on ClinicalTrials.gov with the identification number NCT04180046. NULU primary cell line was derived from a female patient, 36 years old, with GBM grade IV *IDH1* wild type, *MGMT* unmethylated, located in the left parietal region. ZAR primary cell line was derived from a male, 56 years old, with GBM grade IV *IDH1* wild type, and *MGMT* unmethylated, located in the right temporal region. GBM cell lines NULU and ZAR, used in the following experiments, were characterized as previously reported [7]. In detail, primary GBM cells NULU and ZAR were cultured in Dulbecco’s Modified Eagle’s Medium (DMEM) supplemented with 10% Fetal Bovine Serum (FBS), 2 mmol/L-glutamine, 100 IU/mL Penicillin, and 100 μg/mL Streptomycin at 37 °C, 5% CO_2_, and 95% humidity. The medium was changed every 3 days.

### 4.2. Cell Counts

Patient-derived glioblastoma cells NULU and ZAR were seeded in 48-well plates at 1 × 10^4^ cells per well in DMEM supplemented with 10% FBS and cultured at 37 °C in an atmosphere containing 5% CO2. Based on a previous study conducted in our laboratory using the commercial GBM line U87MG [19], as well as the primary lines NULU and ZAR growing in adhesion to the plate, they were treated daily with AE 20 μM and TMZ 10μM as single agents and in combination (AE 20 μM and TMZ 10μM), for 24, 48, and 72 h. For controls, 0.3% DMSO was used. After a PBS washing, cells were detached with 0.25% trypsin, resuspended in 100 μL of medium, and then counted in a Burker chamber.

### 4.3. Cell Viability Assay (MTT)

The long-term effect of AE on cell viability was evaluated by plating 5 × 10^3^ cells in 96-well plates and allowing them to adhere for 12 h. ZAR and NULU GBM cell lines were then treated with AE 20 μM and TMZ 10 μM as single agents and in combination (AE 20 μM and TMZ 10 μM) every 24 h for 24, 48, and 72 h, followed by MTT (3-(4,5-dimethylthiazol-2-yl)-2,5-diphenyltetrazolium bromide) (Sigma–Aldrich, St. Louis, MO, USA). Additionally, 0.3% DMSO was used as a control. Briefly, cultured cells were spiked with 5 mg/mL of MTT in 100 μL of DMEM. Formazan crystals, formed by the oxidation of tetrazolium salts, were dissolved in acidic isopropanol solution, and the absorbance of the solution was determined at 595 nm using an AMR-100T Microplate Reader.

### 4.4. Clonogenic Assay

The colony formation assay was performed by plating 1 × 10^3^ cells per well in triplicate on 6-well plates for 48 h. ZAR and NULU cells were treated with AE 20 μM and TMZ 10 μM as single agents and in combination (AE 20 μM and TMZ 10 μM) and DMSO 0.3% as control for 24 h. Subsequently, the medium was replaced every 3 days for 14 days. For the evaluation of the clonogenic potential of the ZAR and NULU primary GBM lines and the determination of the number of colonies formed under the different treatment conditions, the cells were fixed with a 4% paraformaldehyde solution for 5 min, washed with PBS, and stained with 0.05% crystal violet for 30 min. The stained colonies were counted under an optical microscope at 40× magnification.

### 4.5. Wound Healing Assay

Cell motility was assessed by plating ZAR and NULU GBM cells (5 × 10^4^/well) in 6-well plates. At 90% confluency, a “scratch” was gently created across the cell monolayer using sterile 100 μL tips. The detached cells were washed off with PBS. The cells were treated, respectively, with AE 20 μM and TMZ 10 μM as single agents and in combination (AE 20 μM and TMZ 10 μM), and DMSO 0.3% was used for control. The ability of the cells to close the “scratch” was analyzed by acquiring images under the Evos FL microscope (Life Technologies, Thermo Fisher Scientific, San Jose, CA, USA) for each time point (T0, T24 h, T48 h, T72 h, and T96 h). The scratch area was quantified by image analysis with ImageJ 1.52 software National Institutes of Health USA.

### 4.6. Western Blot

ZAR and NULU primary GBM lines were plated at a density of 0.5 × 10^6^ cells in 60 mm diameter plates in DMEM with 10% FBS and 1% pen/strep and allowed to adhere. The medium was then replaced with DMEM with 0.5% FBS for 48 h for cell cycle synchronization. Treatments were performed every 24 h with AE 20 μM and TMZ 10 μM, as single agents and in combination (AE 20 μM and TMZ 10 μM), DMSO 0.3% as control, and cells were collected after 72 h of treatment.

Proteins were extracted from ZAR and NULU primary GBM lines and treated, as described, in a Triton X-100 lysis buffer (10 mM Tris-HCL, 1 mM EDTA, 150 mM NaCl, 1% Triton X-100, 1 mM NaF, 1 mM Na_4_P_2_O_7_, 1 mM Na_3_VO_4_, and 1× protease inhibitors). Proteins (15 μg) were separated via sodium dodecyl sulfate-polyacrylamide gel electrophoresis (SDS-PAGE) and were transferred to PVDF membranes via electroblotting. The membranes were incubated for 1 h at room temperature in 5% milk diluted in a solution of TBS and Tween-20 (TBS-T) and then incubated overnight at 4 °C with polyclonal anti-MGMT (Cell Signaling, 1:1000), NF-κB p65 (Cell Signaling, 1:1000), and NF-κB p50 (Cell Signaling, 1:500) primary antibodies (Cell Signaling, 1:1000). After 3 washes with TBS-T, the membranes were incubated with rabbit secondary antibodies conjugated to peroxidases (HRP). Proteins were detected by chemiluminescence using ECL Western blotting (Amersham, GE Healthcare Life Science, Chicago, IL, USA). The signals were detected by a digital scanner and quantified with Image Lab 6.1 Software (Bio-Rad Laboratories, Inc., Hercules, CA, USA). For protein normalization, each membrane was then incubated with a mouse monoclonal antibody anti-β-actin (1:100,000, Santa Cruz Biotechnology, Santa Cruz, CA, USA).

### 4.7. Reverse Transcription of RNA

The mRNA levels of genes involved in DNA-damage-induced responses were assessed using quantitative real-time PCR. Briefly, NULU GBM cells seeded on 6-well plates (4.5 × 10^5^ cells/well) were incubated for 24 h with AE 20 μM, TMZ 10 μM, and the two substances combined. The cells cultured without the treatments were used as controls. Then, cells were washed twice with PBS, detached, and collected by centrifugation (200× *g* for 5 min). Total RNA was extracted using the Total RNA Mini Kit (A&A Biotechnology, Gdansk, Poland), combining the standard TRIzol and column-based methods, while cDNA was synthesized using the Transcriba Kit (A&A Biotechnology, Gdansk, Poland), both according to the manufacturer’s protocol. The concentration of RNA was measured using a sensitive single-tube fluorimeter for fluorescence-based quantitation of nucleic acids and proteins. The obtained value was 90 ng/μL. cDNA was amplified using the predesigned gene-specific primers (*BRCA1*, *ATM*, and *DNA-PK*) (Table 1) with a sensitive Real-Time 2xHS-PCR SYBR Master Mix (A&A Biotechnology, Poland) in the Stratagene MX3005P QPCR System (Agilent Technologies, Inc. Santa Clara, CA, USA). Real-time PCR was performed in a 20-μL reaction volume according to the manufacturer’s instructions. Glyceraldehyde 3-phosphate dehydrogenase (*GAPDH*) was used as a reference gene. Real-time PCR cycles were run using the following thermal cycling profile: initial denaturation at 95 °C for 2 min, 40 cycles of denaturation at 95 °C for 5 s, annealing at 60 °C for 30 s, and extension at 72 °C for 30 s. The expression levels of the tested genes were determined using the 2^−∆∆CT^ method. Checking for the proper size of any PCR reaction in this study was performed with electrophoresis of 10 μL PCR products on 2% agarose gels along with MW markers, staining with ethidium bromide and visualization under ultraviolet (UV) light.

### 4.8. Statistical Analysis

Data were expressed as mean ± SEM of three individual experiments; statistical significance was determined by a one-way ANOVA test, Dunnett’s multiple comparison test, and Student *t*-test, as well as Bonferroni’s multiple comparison test, considering *p*-values < 0.05 statistically significant according to GraphPad Prism. * indicates the significance of all groups with respect to the control group (not treated), while # indicates the significance of all groups with respect to the TMZ group.

### 4.9. Determination of the Half-Maximal Inhibitory Concentration (IC50) of AE in ZAR Cells

To estimate the IC50 values of AE in ZAR at 72 h, cells were plated in 96-well plates (5 × 10^3^ cells/well) and treated with AE at different concentrations (0, 5, 10, 20, 30, 40, and 60 μM) via only one induction. The IC50 values were calculated using GraphPad Prism (GraphPad Software Inc., San Diego, CA, USA).

## 5. Conclusions

This study introduces a novel strategy for the adjuvant treatment of GBM. It is interesting to note that AE, a naturally occurring anthraquinone derived from aloe leaves, has been demonstrated to enhance the impact of TMZ alone in controlling the development of GBM cells in vitro, particularly in cells with unmethylated *MGMT* promoters, which exhibit a drug-resistant phenotype. Additionally, AE demonstrates the capacity to inhibit colony formation and reduce cell migration.

## Figures and Tables

**Table 1:** Gene-specific primers were used for *BRCA1*, *ATM*, and *DNA-PK* reverse transcriptase.

Oligo Name	Type	Sequence 5′–3′
***GAPDH***	forward	AGAAAATCTGGCACCACACC
	reverse	GGGGTGTTGAAGGTCTCAAA
***BRCA1***	forward	ACTGCAGCCAGCCACAGGTA
	reverse	TGACCAGGACAGTAGAAGGA
***ATM***	forward	TTTACCTAACTGTGAGCTGTCTCCAT
	reverse	ACTTCCGTAAGGCATCGTAACAC
***DNA-PK***	forward	CCAGCTCTTACGCTCTGATATG
	reverse	CAAACGCATGCCCAAAGTC

## Data Availability

The raw data supporting the conclusions of this article will be made available by the authors upon request, without undue reservation.

## Supplementary Materials

The following supporting information can be downloaded at https://www.mdpi.com/article/10.3390/molecules28166024/s1. Figure S1: IC50 evaluation of ZAR cells at 72 h of treatment with AE. Supplementary Data S1 and S2: Migration of the primary line glioblastoma cells ZAR in response to AE 20 μM alone and in combination with TMZ 10 μM. Representative images of the scratch test in the ZAR cell line, exposed to treatment with AE 20 μM, single and in combination with TMZ 10 μM at 0, 24, and 96 h, under the Evos FL microscope (4× magnification). Controls were treated with 0.3% DMSO. The graph shows the quantification of the scratch test as a percentage of closure of the selected area. For all experiments, the reported values are the mean ± SEM of 3 independent determinations. One-way ANOVA, Bonferroni’s multiple comparison test, and *p*-value < 0.05 are significant. According to GraphPad Prism 7 software, * *p*-values between 0.01 and 0.05; ** *p*-value between 0.001 and 0.01 (with respect to the control); *** *p*-values between 0.0001 and 0.001 (compared to the control), ## *p*-value (compared to TMZ); Effect of AE on ZAR clonogenic potential. (A) Representative ZAR line clonogenic potential assay and the impact of AE 20 μM alone and in combination with TMZ 10 μM on the capacity to form colonies.
