# Supplementary material for: Aloe-Emodin Overcomes Anti-Cancer Drug Resistance to Temozolomide and Prevents Colony Formation and Migration in Primary Human Glioblastoma Cell Lines NULU and ZAR
Source: Molecules. 2023 Aug 11;28(16):6024. doi: 10.3390/molecules28166024 (PMC10458156; doi:10.3390/molecules28166024)
Supplement: Supplementary file 1 [file molecules-28-06024-s001.zip › molecules-2452293-supplementary.pdf]

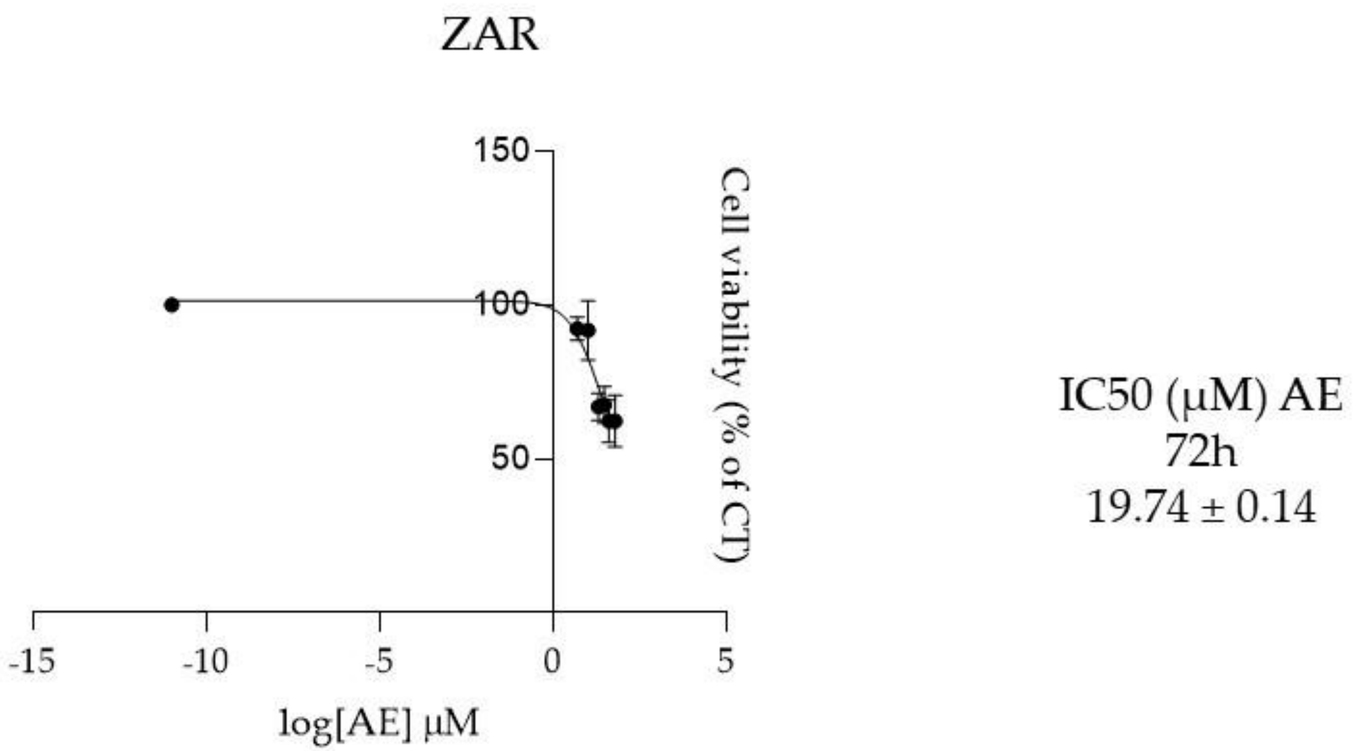

Figure S1. IC50 evaluation of ZAR cells at 72 h of treatment with AE.

Supplementary Data S1

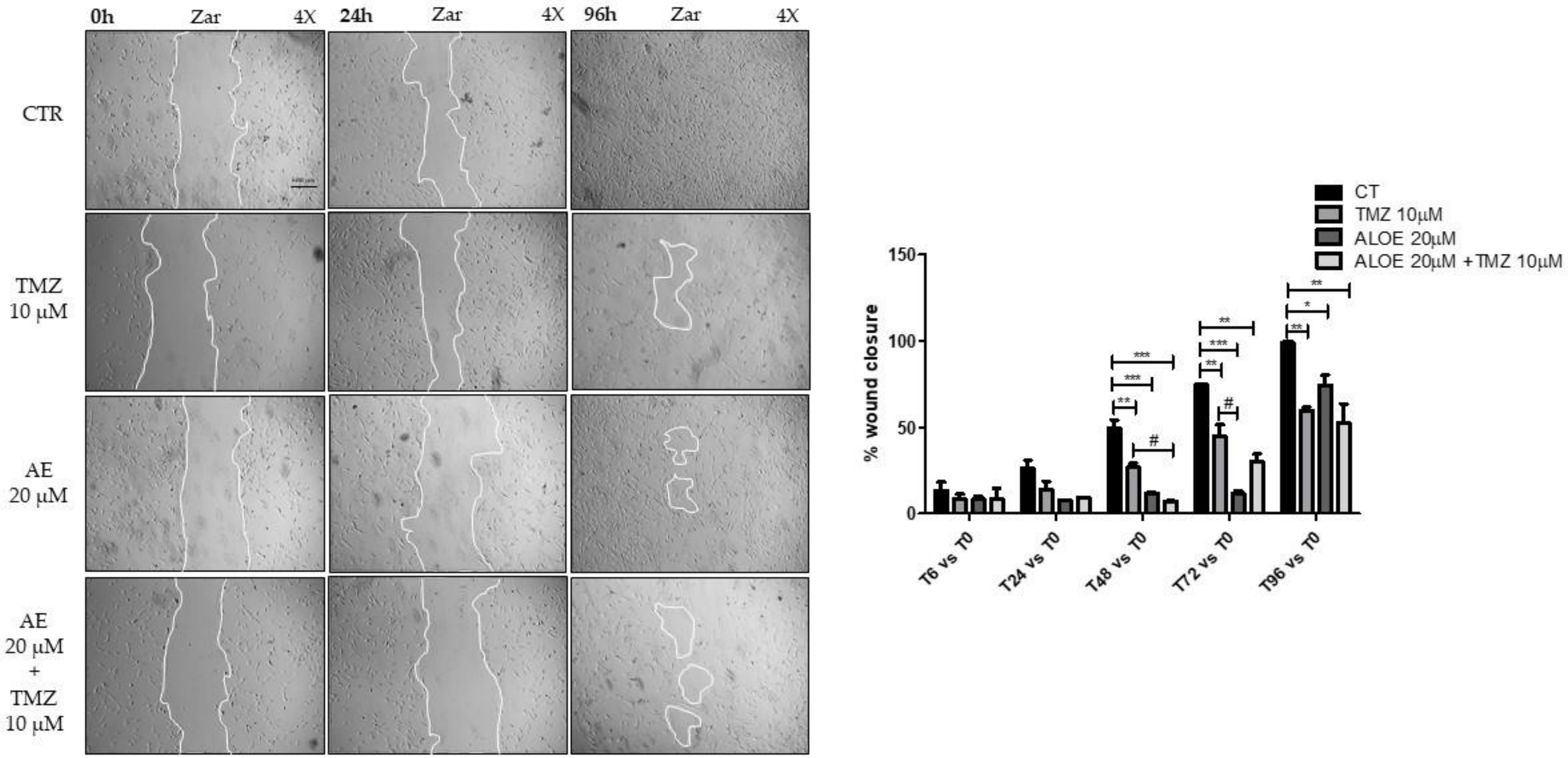

Migration of the primary line glioblastoma cells ZAR in response to AE 20 µM alone and in combination with TMZ 10 µM. Representative images of the scratch test in the ZAR cell line, exposed to treatment with AE 20 µM, single and in combination with TMZ 10 µM at 0, 24, and 96 h, under the Evos FL microscope (4× magnification). Controls were treated with 0.3% DMSO. The graph shows the quantification of the scratch test as a percentage of closure of the selected area. For all experiments, the reported values are the mean ± SEM of 3 independent determinations. One-way ANOVA, Bonferroni's multiple comparison test, and *p*-value < 0.05 are significant. According to GraphPad Prism 7 software, * *p*-values between 0.01 and 0.05; ** *p*-value between 0.001 and 0.01 (with respect to the control); *** *p*-values between 0.0001 and 0.001 (compared to the control), ## *p*-value (compared to TMZ).

Supplementary Data S2

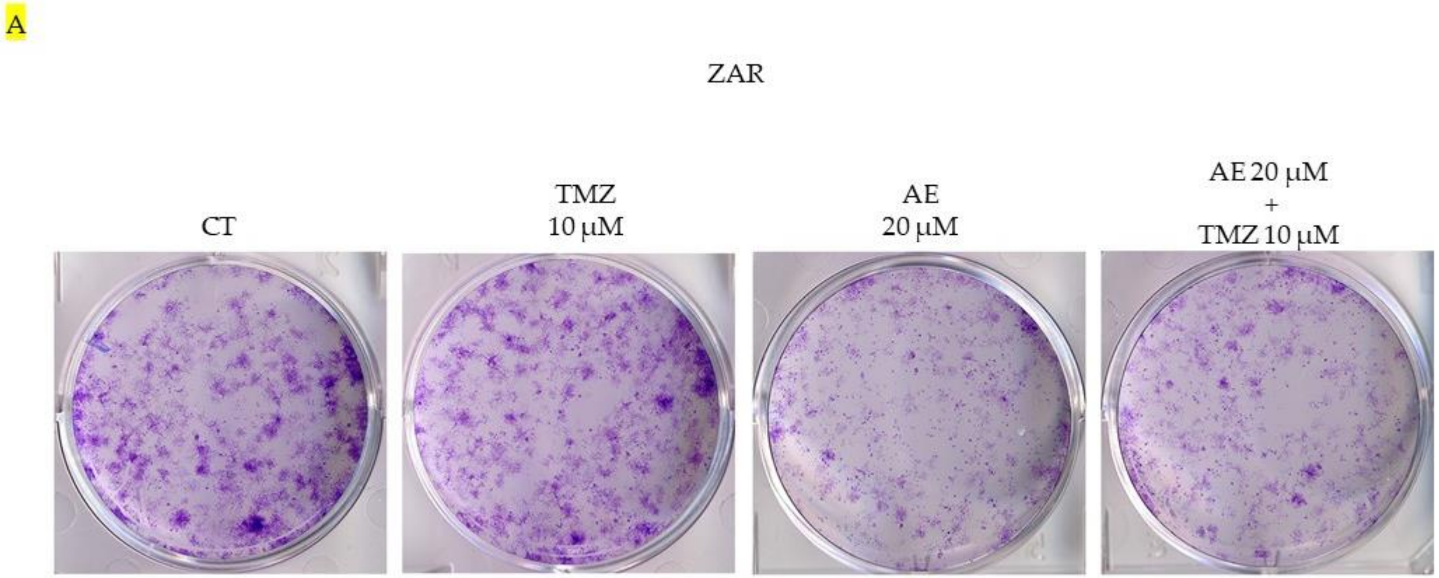

Effect of AE on ZAR clonogenic potential. (**A**) Representative ZAR line clonogenic potential assay and the impact of AE 20 μM alone and in combination with TMZ 10 μM on the capacity to form colonies.
